# Abatacept increases T cell exhaustion in early RA individuals who carry HLA risk alleles

**DOI:** 10.3389/fimmu.2024.1383110

**Published:** 2024-04-08

**Authors:** Sarah Alice Long, Virginia S. Muir, Britta E. Jones, Valerie Z. Wall, Alyssa Ylescupidez, Anne M. Hocking, Stephan Pribitzer, Jerill Thorpe, Bryce Fuchs, Alice E. Wiedeman, Megan Tatum, Katharina Lambert, Hannes Uchtenhagen, Cate Speake, Bernard Ng, Alexander T. Heubeck, Troy R. Torgerson, Adam K. Savage, Michael A. Maldonado, Neelanjana Ray, Vadim Khaychuk, Jinqi Liu, Peter S. Linsley, Jane H. Buckner

**Affiliations:** ^1^ Center for Translational Immunology, Benaroya Research Institute at Virginia Mason, Seattle, WA, United States; ^2^ Center for Systems Immunology, Benaroya Research Institute at Virginia Mason, Seattle, WA, United States; ^3^ Center for Interventional Immunology, Benaroya Research Institute at Virginia Mason, Seattle, WA, United States; ^4^ VA National Rheumatology Program, Specialty Care Program Office, Washington, DC, United States; ^5^ Rheumatology Section, VA Puget Sound Health Care System, Seattle, WA, United States; ^6^ Department of Medicine, Division of Rheumatology, University of Washington, Seattle, WA, United States; ^7^ Allen Institute for Immunology, Seattle, WA, United States; ^8^ Bristol Myers Squibb, Princeton, NJ, United States

**Keywords:** T cell exhaustion, autoimmunity, HLA risk alleles, rheumatoid arthritis, abatacept

## Abstract

Exhausted CD8 T cells (T_EX_) are associated with worse outcome in cancer yet better outcome in autoimmunity. Building on our past findings of increased TIGIT^+^KLRG1^+^ T_EX_ with teplizumab therapy in type 1 diabetes (T1D), in the absence of treatment we found that the frequency of TIGIT^+^KLRG1^+^ T_EX_ is stable within an individual but differs across individuals in both T1D and healthy control (HC) cohorts. This TIGIT^+^KLRG1^+^ CD8 T_EX_ population shares an exhaustion-associated EOMES gene signature in HC, T1D, rheumatoid arthritis (RA), and cancer subjects, expresses multiple inhibitory receptors, and is hyporesponsive *in vitro*, together suggesting co-expression of TIGIT and KLRG1 may broadly define human peripheral exhausted cells. In HC and RA subjects, lower levels of EOMES transcriptional modules and frequency of TIGIT^+^KLRG1^+^ T_EX_ were associated with RA HLA risk alleles (DR0401, 0404, 0405, 0408, 1001) even when considering disease status and cytomegalovirus (CMV) seropositivity. Moreover, the frequency of TIGIT^+^KLRG1^+^ T_EX_ was significantly increased in RA HLA risk but not non-risk subjects treated with abatacept (CTLA4Ig). The DR4 association and selective modulation with abatacept suggests that therapeutic modulation of T_EX_ may be more effective in DR4 subjects and T_EX_ may be indirectly influenced by cellular interactions that are blocked by abatacept.

## Introduction

Chronic antigen exposure leads to the progressive differentiation of exhausted CD8 T cells (T_EX_) that are functionally, transcriptionally, and epigenetically distinct from effector CD8 T cells (1). T_EX_ progressively lose inflammatory cytokine production from precursor and early T_EX_ states to terminal states. This loss of pro-inflammatory function is mediated, in part, by constitutive expression of multiple inhibitory receptors (e.g., PD-1, TIGIT, TIM3). In cancer and chronic viral settings, where lytic properties of CD8 T cells are required to clear the tumor or virus, an increase in T_EX_ abundance that are terminally dysfunctional is associated with worse outcome (2). Likewise, a greater abundance of T_EX_ in cancer is associated with worse outcome and progression can be reversed by therapeutically depleting terminal T_EX_ or reinvigorating early T_EX_ with checkpoint inhibitors (3–5).

Although autoimmunity may involve chronic antigen exposure like cancer and chronic viral infections, less is known about T_EX_ in the context of autoimmune disease (6, 7). As opposed to reduced T_EX_ being beneficial in cancer, reduced T_EX_ has been associated with disease progression and increased severity in some autoimmune diseases including systemic lupus erythematosus (SLE), antineutrophil cytoplasmic antibody-associated vasculitis, and type 1 diabetes (T1D) (8–11). Conversely, elevated T_EX_ has been associated with better response to therapy in individuals with autoimmune disease; Specifically, elevated T_EX_ following treatment of T1D was associated with better response to two T cell targeted therapies, teplizumab (anti-CD3) (12, 13) and alefacept (LFA3Ig) (14). T_EX_ levels do not differ at baseline, but the T_EX_ that expand following therapy in T1D co-express inhibitory receptors including TIGIT, PD-1, and KLRG1, and share an EOMES gene signature that overlaps with exhaustion and differs from senescence (14), suggesting an exhausted-like phenotype (12, 13). Despite these findings, it remains to be determined whether T_EX_ are qualitatively similar across diseases and which factors may contribute to variation in T_EX_ levels across subjects. This lack of clarity is due, in part, to variability in the measures used to define T_EX_ across studies and disease specific variability in co-factors that contribute to T_EX_.

In this current study, we address these gaps in knowledge by leveraging existing cross-sectional and longitudinal cohorts as well as existing datasets from recent clinical trials. Comparison across cohorts was facilitated by defining a broad and common human peripheral blood T_EX_ population that co-expresses the inhibitory receptors TIGIT and KLRG1 and is associated with an EOMES transcriptional signature. Looking across studies, we determined that the frequency of TIGIT^+^KLRG1^+^ T_EX_ is influenced by a genetic component. Specifically, reduced T_EX_ frequencies are associated with the human leukocyte antigen (HLA) class II alleles DRB1*0401, 0404, 0405, 0408, and 1001 associated with risk of RA in both healthy individuals and individuals with RA. Moreover, this relationship was also evident in the setting of clinical trials where TIGIT^+^KLRG1^+^ T_EX_ selectively increased in individuals carrying RA HLA risk alleles but not in carriers of non-risk alleles after treatment with abatacept (CTLA4Ig). Together these data suggest that HLA or linked genes contribute to the level of T_EX_ in a manner that is modulated by abatacept.

## Methods

### Ethics statement

All subjects in the longitudinal healthy control cohort and the whole blood RNA-seq cohort gave written informed consent in accordance with the Declaration of Helsinki, the IRB-approved protocols at the Benaroya Research Institute at Virginia Mason (IRB07109), and the VA Puget Sound Health Care System (MIRB#00755). The clinical trial cohorts were approved by independent IRBs at each participating clinical site, as described in the original clinical trial reports (15–17). Participants in each of these trials also provided informed consent prior to participation.

### Study design

The phenotype, frequency, function, and modulation of T_EX_ were assessed using complementary assays and cohorts (**Supplementary Figure 1**, **Table 1**). Cross-sectional samples were used from T1D, RA, and renal cancer carcinoma (RCC) patients with age- and sex-matched health controls (HC). Longitudinal samples were analyzed from HC subjects and published clinical trials (15, 17, 18). Whole blood transcriptional analyses were performed from tempus tube collections. For all cellular analyses, peripheral blood mononuclear cells (PBMCs) were isolated from whole blood and cryopreserved until used. Additional transcriptional analyses were performed on sorted populations from PBMCs. When assessing the influence of age on T_EX_ in HCs, male and female subjects were selected for even representation across all ages. All assays were run and analyzed in a blinded manner, and staining batches included an internal control.

**Table 1:** Longitudinal and clinical cohorts.

Cohort Name	Figure	Cohort Type	Subject Type	Number of subjects^a^	Age (range, mean)	% Caucasian	% Female	% RA HLA risk	% CMV sero-positive	Primary reference
Longitudinal T1D (L-T1D)	1A	Longitudinal	T1D	66	8.5-34.0, 17.8	89.4	33.3	NA	18.5	19-22
Longitudinal HC (L-HC)	1B	Longitudinal	HC	99	25.0 – 65.0, 44.3	86.9	56.6	NA	43.4	This manuscript
Cross-sectional HC (C-HC)	4A	Cross-sectional	HC	114	21.0 – 81.0, 47.9	87.1	63.8	50	46	This manuscript
Cross-sectional RA (C-RA)	4A	Cross-sectional	RA	97	25.0 – 86.0, 57.8	75.7	64	75.7	51.8	This manuscript
Cross-sectional HC (C-HC)^b^	4B	Cross-sectional	HC	10^b^	21.0 – 66.0, 47.7	100	60	50	0	This manuscript
Cross-sectional RA (C-RA)^b^	4B	Cross-sectional	RA	10^b^	39.0 – 74.0, 57.1	100	69	50	0	This manuscript
Clinical trial T1D (CT-T1D)	5A	Longitudinal	T1D risk	32	8.0 – 45.0, 15.3	92.1	43.3	68.8	20.7	15
Clinical trial RA (CT-RA)	5B, C	Longitudinal	RA	29	28.0 – 71.0, 48.2	93.1	75.9	51.7	Not available	16

**Figure 1:**
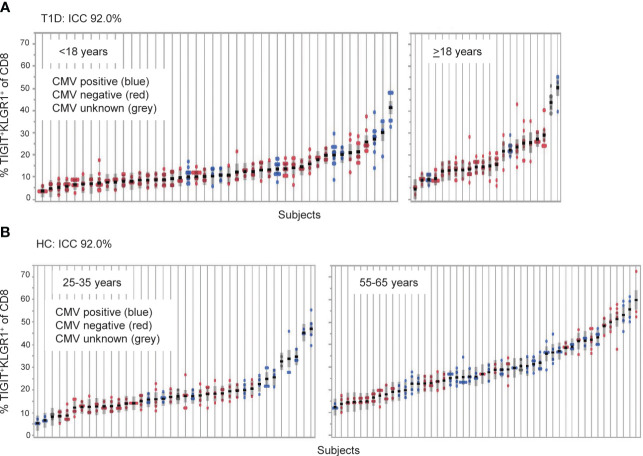
TIGIT^+^KLRG1^+^ CD8 T cells are a stable cell type that varies across individuals. TIGIT^+^KLRG1^+^ CD8 T cells were measured by flow cytometry in longitudinal samples from **(A)** Individuals with type 1 diabetes (T1D; *n* = 66) and **(B)** healthy control subjects (HC; *n* = 99). T1D samples were collected at 6-month intervals over 2 years. HC samples were collected over a median of 8.7 months (inter-quartile range 7.2 to 14.2). Multiple samples (data points) isolated from individual subjects, each shown on a line, are graphed for both cohorts. Individuals are ordered by mean % TIGIT^+^KRLG1^+^ and annotated for CMV seropositivity by color. ICC, Intraclass correlation coefficient.

HC, healthy control; RA, Rheumatoid Arthritis, T1D, type 1 diabetes; NA, not available; RA HLA risk, DR0401, 0404, 0405, 0408, 1001.

a number of subjects with HLA and flow cytometry data at all time points studied.

b subset of cohort in **Figure 1A**.

### Cohort descriptions

**Figure 2:**
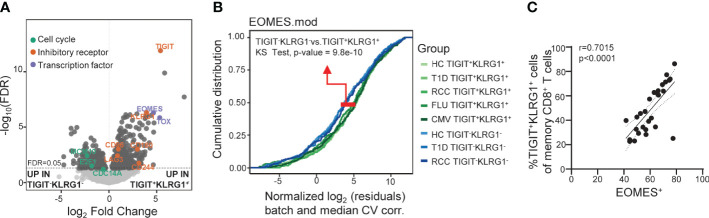
Across disease settings, co-expression of TIGIT and KLRG1 marks memory CD8 T cells with an EOMES-associated transcriptional signature. **(A)** Bulk RNA-seq data from sorted TIGIT^+^KLRG1^+^ and TIGIT^-^KLRG1^-^ CD8 memory (not CD45RA^+^CCR7^+^ naïve) T cells isolated from healthy control subjects (HC) (*n* = 4) following 16-hour anti-CD3/CD28 stimulation. Selected markers of cell cycle, inhibitory receptor and transcription factor expression are annotated. **(B)** Comparison of sorted TIGIT^+^KLRG1^+^ and TIGIT^-^KLRG1^-^ memory CD8^+^ T cells across multiple disease settings: age- and gender-matched HC; type 1 diabetes (T1D); renal cell carcinoma (RCC); cytomegalovirus infection (CMV-pentamer positive cells); and influenza infection (FLU-pentamer positive cells). **(C)** Correlation of EOMES protein expression and TIGIT^+^KLRG1^+^ in memory (not CD45RA+CCR7+ naïve) CD8 T cells in HC (*n* = 29), Spearman test with 95% confidence interval (dotted lines). Gating for sorts and analyses are shown in **Supplementary Figure 2**.

**Figure 3:**
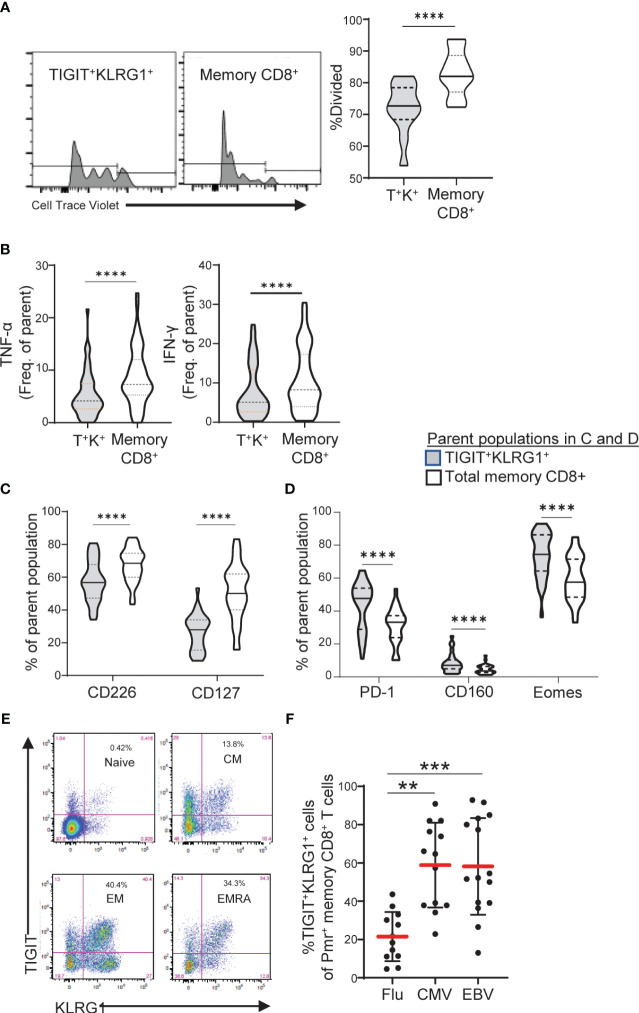
TIGIT^+^KLRG1^+^ memory CD8 T cells are dysfunctional in healthy control subjects and increased in terminal cell subsets and chronic viral reactive cells. **(A)** Proliferation following 3-day anti-CD3/CD28 stimulation of TIGIT^+^KLRG1^+^ (T^+^K^+^) memory cells relative to total memory CD8^+^ T cells from healthy control (HC) subjects (*n* = 12). Proliferation of memory CD45RO^+^ cells was measured by percentage of divided cells using a flow cytometry dye dilution assay. **(B)** Pro-inflammatory cytokine production (TNF-α and IFN-γ) following 24-hour anti-CD3/CD28 stimulation by gated T^+^K^+^ memory cells relative to total memory CD8^+^ T cells in HC subjects (*n* = 56). Cytokine production was measured by intracellular cytokine staining. **(C)** Effector cell surface marker expression (CD226 and CD127) and **(D)** Inhibitory receptor expression in the absence of T cell activation in gated T^+^K^+^ cells relative to total memory CD8^+^ T cells from HC subjects (*n* = 28). Wilcoxon matched-pairs signed-rank test was used in all comparisons. **(E)** Distribution of TIGIT^+^KLRG1^+^ cells within naïve (CD45RO^-^CCR7^+^), central memory (CM: CD45RO^+^CCR7^+^), effector memory (EM: CD45RO^+^CCR7^-^) and RA^+^ effector memory (EMRA: CD45RO^-^CCR7^-^). One representative HC sample shown from **C**. **(F)** TIGIT^+^KLRG1^+^ distribution in a subset of HLA-A2 subjects stained with Flu-, CMV- and EBV-specific Class I Pentamer (Pmr). Kruskal-Wallis test with Dunn’s correction for multiple tests. Gating for memory TIGIT^+^KLRG1^+^ is shown in **Supplementary Figure 2**, gating for activating and inhibitory markers is shown in **Supplementary Figure 4**. **=0.05, ***=0.005, ****=0.0005 p-values.

**Figure 4:**
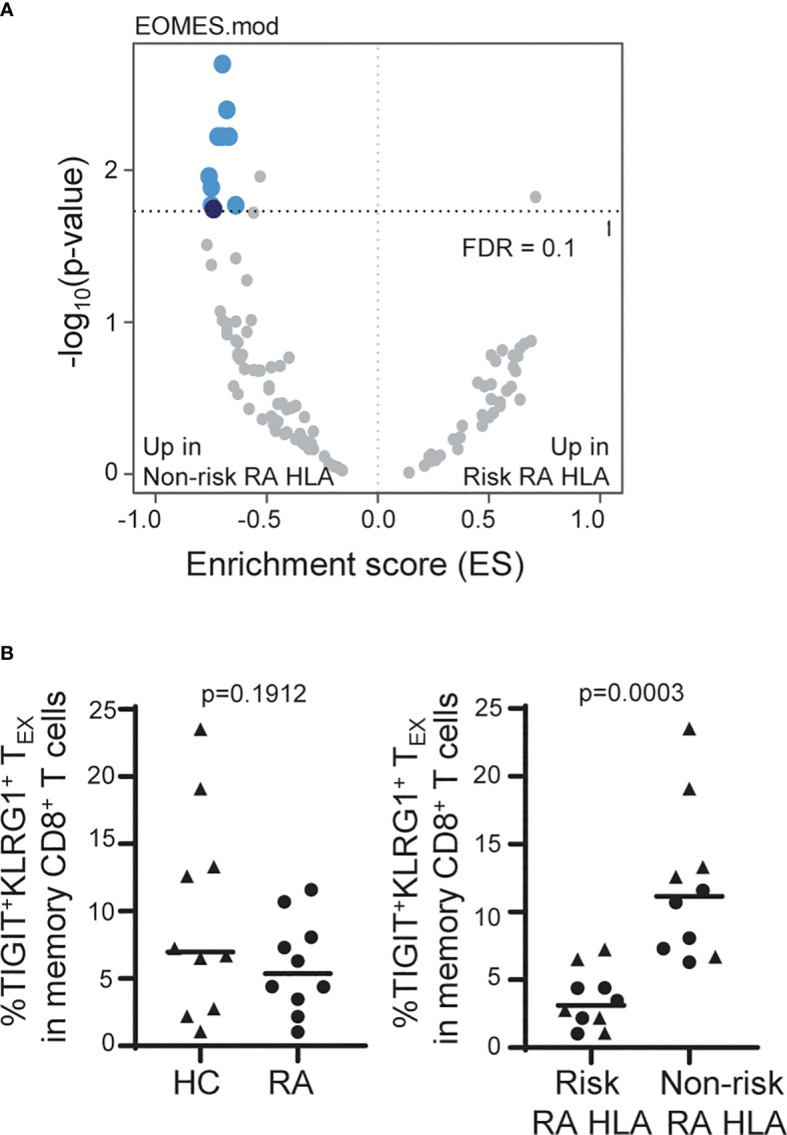
The frequency of TIGIT^+^KLRG1^+^ T_EX_ is influenced by RA HLA risk alleles. **(A)** Whole blood RNA-seq data of age- and sex-matched HC (*n* = 114) and RA (*n* = 97) subjects were parsed by RA-associated HLA risk genotype (DRB1*0401, 0404, 0405, 0408, 1001). Dark blue, EOMES module; light blue, EOMES module overlap; gray, no overlap with EOMES module. **(B)** Frequency of TIGIT^+^KLRG1^+^ memory CD8 T cells in age- and sex-matched HC and RA subjects (*n* = 10/cohort) selected for top or bottom tercile EOMES signature: *Left*, HC versus RA; *Right*, Risk RA HLA versus non-risk RA HLA. Mann-Whitney test. Gating shown in **Supplementary Figure 2**.

**Figure 5:**
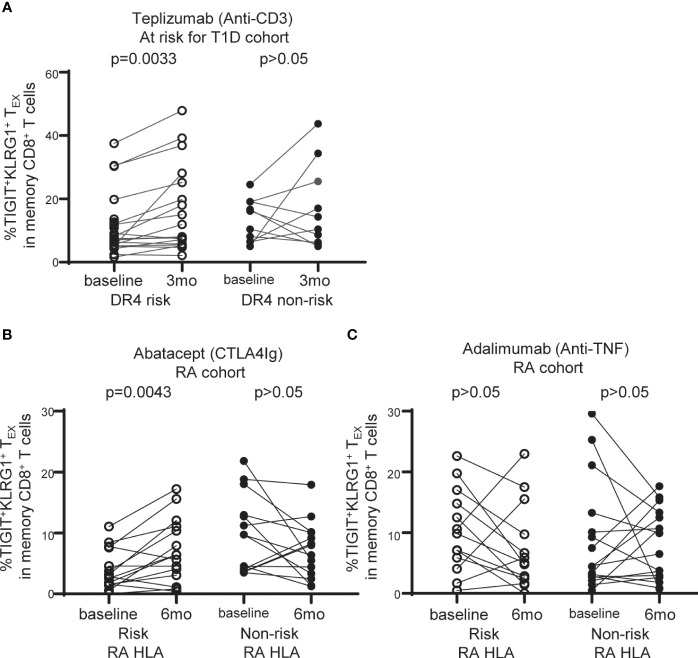
TIGIT^+^KLRG1^+^T_EX_ are selectively increased with abatacept therapy in RA subjects carrying RA HLA risk alleles. **(A)** Frequency of TIGIT^+^KLRG1^+^ T_EX_ in memory CD8^+^ T cell compartment in individuals at risk for T1D treated with teplizumab (anti-CD3) stratified by DR4 risk and DR4 non-risk. **(B)** Frequency of TIGIT^+^KLRG1^+^ T_EX_ in memory CD8^+^ T cell compartment in individuals with new onset rheumatoid arthritis (RA) treated with abatacept (CTLA4-Ig) stratified by risk RA HLA and non-risk RA HLA. **(C)** Frequency of TIGIT^+^KLRG1^+^ T_EX_ in memory CD8^+^ T cell compartment in individuals with new onset RA adalimumab (anti-TNF) stratified by risk RA HLA and non-risk RA HLA. In **B** and **C**, risk RA HLA carried either DRB1*0401, 0404, 0405, 0408, or 1001 and non-risk RA HLA did not. In all three trials, age and CMV status (where available) did not differ between HLA groups. Wilcoxon matched-pairs signed-rank test was used for each risk group in all studies. Gating shown in **Supplementary Figure 2**.

**Table 1** lists cohorts used in this study, including demographics, percentage of RA HLA risk carriers and percentage of cytomegalovirus (CMV)-seropositive subjects. The longitudinal T1D cohort in **Figure 1A** consisted of 66 subjects with recent onset T1D who were placebo arms of Immune Tolerance Network and TrialNet trials (19–22). The longitudinal HC cohort in **Figure 1B** consisted of 99 HC subjects with no personal or family history of autoimmune disease who were recruited through the Sound Life Project led by the Benaroya Research Institute (BRI) in partnership with the Allen Institute for Immunology. The cross-sectional HC, T1D, and RCC cohorts in **Figure 2** were from the BRI Registry and Repository; the HC had no personal history or first-degree relatives with autoimmune disease. The cross-sectional HC cohort in **Figure 3F** consisted of 30 individuals who had no personal history or first-degree relatives with autoimmune disease who were recruited through the BRI Registry and Repository. The whole blood RNA-seq cohort in **Figure 4A** is a cross-sectional cohort consisting of 97 seropositive RA subjects and 114 HC subjects matched for age, sex, and race. The RA subjects carried a diagnosis of RA based on the 2010 American College of Rheumatology criteria, were positive for ACPA and were recruited from the Virginia Mason Medical Center and the VA Puget Sound Health Care System. HC subjects had no first-degree relatives with autoimmune disease and were recruited through the BRI Registry and Repository. The clinical trial cohort in **Figure 5A** was from the teplizumab (anti-CD3) trial in individuals at risk for T1D conducted by the Type 1 Diabetes TrialNet (15) and consisted of 32 subjects. The clinical trial cohort in **Figures 5B, C** was from the Early AMPLE trial (16) and consisted of 29 individuals with new onset RA.

### Transcriptional analyses of CD8 cell subsets

PBMCs from subjects with T1D, RA, RCC and age/gender-matched HC were stimulated with antibodies against CD3 (1 µg/ml plate-bound, UCHT1) and CD28 (2 µg/ml plate-bound, CD28.2) for 16 hours with and sorted for memory (CD45RO^+^) CD8 T cells that either co-expressed KLRG1 and TIGIT or lacked both markers as a comparison population using the cell sorting panel (**Supplementary Table 1**). Sytox Green (1:1000, Invitrogen) was added to samples prior to acquisition to differentiate dead cells. For comparisons across cells of differing antigen specificities, cells from HC were enriched for CD8 T cells following the 16-hour stimulation protocol, then stained with Class I pentamer to identify CMV, Epstein-Bar Virus (EBV), and Flu antigens (**Supplementary Table 1**) as described below.

The indicated populations were sorted directly into SMARTer v3 or SMARTseq v4 lysis reagents (Clontech). Cells were lysed and cDNA was synthesized. After amplification, sequencing libraries were prepared using the Nextera XT DNA Library Preparation Kit (Illumina) according to C1 protocols (Fluidigm). Barcoded libraries were pooled and quantified using a Qubit^®^ Fluorometer (Life Technologies). Single-read sequencing of the pooled libraries was carried out on a HiSeq2500 sequencer (Illumina) for 74 cycles, using TruSeq v3 or v4, and SBS kits (Illumina). Target read depths were ~5-10 million raw reads per sample.

### Characterization of T cell activation/exhaustion by flow cytometry

**Supplementary Table 1** lists antibodies used for each flow cytometry panel, including target, fluorophore, clone, and manufacturer. For assessment of cytokines, cells were treated with anti-CD3 (1 µg/ml plate-bound, OKT3) and anti-CD28 (2 µg/ml plate-bound, CD28.2) for 24 hours or Phorbol-Myristate-Acetate (PMA, Sigma) and Ionomycin (I, Sigma) for 6 hours and Brefeldin A (BioLegend) and Monensin (BioLegend) were each added at 1X for the last 4 hours. Dead cells were detected (Zombie NIR Kit, BioLegend), surface markers were added in brilliant stain buffer (BD Biosciences) for 20 minutes at RT and intracellular markers were detected (30 minutes RT) following permeabilization (FoxP3/Transcription factor staining buffer set, eBioscience, 30 minutes at 4°C). For assessment of antigen-specific phenotype, cells were enriched for CD8 T cells using negative selection (CD8^+^ T cell isolation Kit, Miltenyi) and incubated with dasatinib (50 nM, 250 µl/2 million cells, LC Laboratories) for 8-10 minutes at 37°C prior to staining with 25 µL solution containing 1.5 µL of commercially obtained Class I pentamer (ProImmune) for 15 minutes at 37°C, followed by surface marker detection in the T1D antigen-specific panel (**Supplementary Figure 2**).

### Cell tracking assay

PBMCs were labeled (Cell Trace Violet, Invitrogen), stained with surface markers of the cell sorting panel (**Supplementary Table 1**, **Supplementary Figure 3**) and Sytox Green to differentiate dead cells (1:1000, Invitrogen). PBMCs were sorted using a BD Aria II until 3-5 × 10^3^ CD8 T cells were obtained per condition which co-expressed KLRG1 and TIGIT or lacked both markers. Sorted cells were mixed back into whole PBMCs from the same subject and stimulated with anti-CD3 (1 µg/ml plate-bound, UCHT1). Percent divided were assessed in labeled cells using FlowJo proliferation modeling. Labeled cells were also assessed for changes in KLRG1 and TIGIT expression following stimulation (% Stable = purity of labeled population following stimulation/purity of labeled population at baseline × 100).

### Whole blood RNA-sequencing

RNA isolation, RNA-seq, and pipeline analyses including differential expression (Limma-Voom) and protein-protein networks were performed as described previously (23).

### Single nucleotide polymorphisms association analysis

Whole blood libraries from the 211 HC and RA subjects in **Figure 4A** were TMM normalized and batch corrected for age, percent lymphocytes and percent duplication, a quality metric associated with PC1. SNPs were generated with an Affymetrix Axiom PMRA chip and single nucleotide polymorphisms (SNPs) on chromosome six (containing the HLA region) were selected for study. SNPs exhibiting little variance or frequent missing genotypes were removed from the analysis. The most significant DRB1*04 associated SNP (rs72492350) and an unassociated SNP (Chr6:32183175) were used as phenotypes in separate GSEA analyses (24) with 100-gene modules (25).

### Statistical analyses

A linear mixed-effects model with a random effect for subject was used to calculate intraclass correlation coefficient (ICC), which quantifies the proportion of biomarker variation that is within and between subjects. Age and CMV seropositivity were added as covariates to investigate their association with biomarker frequency. Summary statistics include mean, range, median, and inter-quartile range; 95% confidence intervals are reported where appropriate. Spearman’s correlation coefficients were used for associations, Kolmogorov-Smirnov tests were used for cumulative distribution comparisons, Wilcoxon matched-pairs signed-rank tests were used for paired comparisons, and Mann-Whitney test was used for unpaired comparisons while a Kruskal-Wallis test with Dunn’s correction for multiple tests was used for multiple unpaired comparisons. All *P* values < 0.05 were considered significant.

## Results

### The frequency of TIGIT^+^KLRG1^+^ T_EX_ varies more across than within subjects

We previously reported that co-expression of TIGIT and KLRG1 marked CD8^+^ T cells that had phenotypic and functional features of exhaustion, including an EOMES signature, and that these cells expanded following teplizumab (anti-CD3) therapy in individuals with T1D (12, 13). To address variation of TIGIT^+^KLRG1^+^ CD8 T cells in the absence of therapy, we first investigated the stability of these cells *in vivo* in a T1D cohort (**Table 1**, **Supplementary Figure 1**) measuring the proportion of TIGIT^+^KLRG1^+^ CD8^+^ T cells at multiple time points over two years (**Figure 1A**). We found that the frequency of TIGIT^+^KLRG1^+^ CD8 T cells varied little within T1D subjects over two years (mean within-subject range 8.2% [95% CI: 6.9-9.5]) but varied greatly between T1D subjects with a mean frequency range of 2.9% to 50.6%. To confirm that this stability is not unique to T1D, we measured the proportion of TIGIT^+^KLRG1^+^ CD8^+^ T cells at multiple time points over two years in a HC cohort (**Table 1**, **Figure 1B**). We found that the frequency of TIGIT^+^KLRG1^+^ CD8 T cells also varied little within HC over time (mean within-subject range 6.5% [95% CI: 5.6-7.4]) while the mean frequency ranged from 4.2 to 59.8%. Lack of variation within subjects is supported by high intraclass correlation coefficient (ICC) values (83.7% and 92.0%, respectively), a measure comparing variability within versus across subjects.

A known contributor to increased T cell exhaustion in an individual is age and chronic viral infection (1). In both the HC and T1D cohorts (**Figure 1**), increasing years of age (effect of 0.32 [95% CI: 0.20, 0.45], *P* = <0.0001) and CMV seropositivity (effect of 3.67 [95% CI: 2.06, 5.28], *P* = <0.0001) were significantly associated with TIGIT^+^KLRG1^+^ CD8 T cell frequency in a linear mixed-effects model. However, disease status did not have a significant effect (*P* = 0.65, fixed effect test) and variance contributed by age and CMV status were significant but not robust (age effect, 0.32, CMV effect, 3.67) suggesting that other factors also contribute to TIIGT^+^KLRG1^+^ variation across subjects.

We also investigated the stability of TIGIT^+^KLRG1^+^ T_EX_ *in vitro* using an *in vitro* assay system designed to track the frequency of TIGIT^+^KLRG1^+^ T_EX_ cells that maintain co-expression of TIGIT and KLRG1 upon activation. Specifically, memory TIGIT^+^KLRG1^+^ cells were sorted and labelled with a cell trace dye to identify them as TIGIT+KLRG1+ prior to activation. Sorted cells were then mixed with autologous PBMCs before activation with anti-CD3/anti-CD28 antibodies (**Supplementary Figure 3A**). Labelled cells were monitored over time for maintenance of TIGIT and KLRG1 expression. Measuring maintenance of this phenotype, we found that TIGIT^+^KLRG1^+^ CD8 T cells were stable for 8 days following anti-CD3/CD28 activation (**Supplementary Figure 3B**).

### TIGIT and KLRG1 co-expression marks EOMES^+^CD8^+^ T_EX_ across human diseases

We previously reported that co-expression of TIGIT and KLRG1 marked CD8^+^ T cells that had phenotypic and functional features of exhaustion, including an EOMES signature, and that these cells expanded following teplizumab (anti-CD3) therapy in individuals with T1D (12–14). To expand the functional characterization of TIGIT^+^KLRG1^+^ CD8 T cells in the absence of therapy, we compared the transcriptome of TIGIT^+^KLRG1^+^ memory CD8^+^ T cells and TIGIT^-^KLRG1^-^ memory CD8^+^ T cells from HC. Similar to our previous finding in the setting of T1D and immunotherapy (12), TIGIT^+^KLRG1^+^ memory CD8^+^ T cells had increased expression of T cell exhaustion markers including the transcription factor TOX and inhibitory receptors (i.e., LAG-3, CD160, CD244), and reduced expression of cell cycle genes (**Figure 2A**, **Supplementary Table 2**). To determine whether TIGIT^+^KLRG1^+^ memory CD8^+^ T cells are similar across disease settings, we compared EOMES module expression in sorted TIGIT^+^KLRG1^+^ memory CD8^+^ T cells and TIGIT^-^KLRG1^-^ memory CD8^+^ T cells from HC, individuals with T1D, and individuals with RCC; RCC was included because cancer is a setting where exhaustion is expected (1). We also included TIGIT^+^KLRG1^+^ CD8^+^ T memory cells sorted from both acute (influenza (FLU) and chronic (CMV) viral-specific T cells identified using pentamer staining (**Supplementary Figure 2**). Across all disease settings tested, TIGIT^+^KLRG1^+^ memory CD8^+^ T cells differed from memory CD8^+^ T cells lacking TIGIT and KLRG1 expression (K-S test, *P* = 9.8e-10) (**Figure B**).

We assessed similarity of the TIGIT^+^KLRG1^+^ EOMES signature with other published signatures of T_EX_ identified across disease settings using cumulative distribution function (CDF) curves, asking whether other published signatures can discriminate TIGIT^+^KLRG1^+^ cells from TIGIT^-^KLRG1^-^ cells. Published T_EX_ signatures included four murine T_EX_ subsets (3), common human cancer T_EX_ signatures (26) and the exhaustion-associated EOMES module that we previously identified in T1D subjects treated with teplizumab (anti-CD3) (12). Given that TIGIT and KLRG1 co-expression were identified in peripheral blood of T1D subjects, the T1D EOMES signature (12) best discriminated transcriptional profiles of TIGIT^+^KLRG1^+^ and TIGIT^-^KLRG1^-^ populations (K-S test, *P* = 9.8e-10). Terminal T_EX_ signatures from the mouse and cancer data sets were also more similar to TIGIT^+^KLRG1^+^ cells (K-S test, *P* = 4.3e-03 and *P* = 9e-06, respectively). Moreover, we confirmed that EOMES protein expression correlates with co-expression of TIGIT and KLRG1 on memory CD8 T cells from HC using flow cytometry (Spearman test: r = 0.7015) (**Figure 2C**). Together, these data suggest that the TIGIT^+^KLRG1^+^ CD8^+^ T cell population is primarily composed of T_EX_ and is present in the peripheral blood in healthy individuals, individuals with autoimmune disease, and cancer.

### TIGIT^+^KLRG1^+^ memory CD8 T cells exhibit reduced effector function

To demonstrate that TIGIT^+^KLRG1^+^ memory CD8^+^ T cells are functionally exhausted and display reduced proliferation and cytokine production, we compared HC TIGIT^+^KLRG1^+^ memory CD8^+^ T cells to the total memory CD8^+^ T cell population which includes all memory CD8^+^ T cell subsets (**Supplementary Figure 4**). Compared to total memory CD8, TIGIT^+^KLRG1^+^ memory CD8^+^ T cells divided fewer times (**Figure 3A**) and produced lower levels of TNF-α and IFN-γ upon T cell receptor stimulation (**Figure 3B**). This reduced effector function corresponded with phenotypic features of exhausted cells. Markers of effector function (CD127, CD226) were reduced, while inhibitory markers (PD-1, CD160, EOMES) were increased (**Figures 3C, D**). Thus, co-expression of TIGIT and KLRG1 on memory CD8^+^ T cells marks phenotypically and functionally exhausted TIGIT^+^KLRG1^+^ CD8^+^ T cells with reduced effector functions. For simplicity, henceforth, we refer to this population as TIGIT^+^KLRG1^+^ T_EX_.

To confirm that TIGIT^+^KLRG1^+^ T_EX_ are increased in settings previously reported to display increased CD8 T cell exhaustion, we parsed TIGIT^+^KLRG1^+^ T_EX_ by progressive differentiation states and acute or chronic viral specificity. TIGIT^+^KLRG1^+^ cells were present in all subsets of CD8^+^ T cells with the majority being memory cells (**Figure 3E**, **Supplementary Figure 2**). Within TIGIT^+^KLRG1^+^ CD8 T cells, effector memory were the most abundant (61%) with central memory (13%) and CD45RA^+^ effector memory (18%) being next abundant in the same dataset analyzed in **Figure 3E**. Consistent with an increase of T cell exhaustion in chronic as compared to acute viral infections (1), we found increased frequencies of TIGIT^+^KLRG1^+^ T_EX_ in CMV- and EBV-specific T cells identified by pentamer reagents as compared to influenza-specific T cells (**Figure 3F**). Thus, TIGIT^+^KLRG1^+^ T_EX_ can be identified across lineages, but are found primarily in effector cells and settings previously associated with increased T_EX_ (1).

### The frequency of TIGIT^+^KLRG1^+^ T_EX_ is influenced by RA HLA risk alleles

Due to the TIGIT^+^KLRG1^+^ T_EX_ stability within and high variation across subjects, we were able to leverage cross-sectional datasets to explore autoimmune-related factors that influence the frequency of TIGIT^+^KLRG1^+^ T_EX_. We examined whole blood RNA sequencing (RNA-seq) data from a large cohort of age- and sex-matched HC and RA subjects (**Table 1**). While we found transcriptional differences between HC and RA, we also observed enrichment in the expression of genes that comprise the EOMES signature previously associated with CD8 T cell exhaustion (12) (**Figure 2**) when stratifying the combined cohorts by RA HLA autoimmune risk alleles (**Figure 4A**). Specifically, we focused on the HLA DRB1*04 alleles (*0401, 0404, 0405 and 0408) and the closely related DRB1*1001 genes most strongly associated with RA (odds ratios > 4.2) (27), and refer to carriers of these alleles as risk RA HLA and non-carriers as non-risk RA HLA. The HLA distribution for the risk RA HLA subjects is shown in **Supplementary Table 3**. Further investigation showed that the enrichment of EOMES modules in the non-risk RA HLA cohort was not due to CMV positivity since CMV-positive subjects were actually underrepresented (46%) in the non-risk RA HLA cohort as compared to the risk RA HLA cohort (52%). Likewise, the EOMES signature does not appear to be secondary to disease as similar enrichment in the non-risk RA HLA cohort was observed when HC were analyzed separately (**Supplementary Table 4**). Last, complementary SNP association analyses within the HLA-DRB1 locus confirmed decreased RNA-seq EOMES module association with risk RA HLA alleles (**Supplementary Figure 5**). Together, these findings support the association of an EOMES signature with the lack of RA HLA risk.

To determine whether the composition of the EOMES signatures in carriers of risk and non-risk RA HLA differ, we compared EOMES module expression in TIGIT^+^KLRG1^+^ memory CD8^+^ T cells isolated from HC carriers of risk and non-risk RA HLA. As in **Figure 2B**, we found the TIGIT^+^KLRG1^+^ cells isolated from both risk and non-risk RA HLA subjects were more similar to each other than their TIGIT^-^KLRG1^-^ counterparts (**Supplementary Figure 6A**). Risk and non-risk RA HLA TIGIT^+^KLRG1^+^ memory CD8 T cells also shared interconnected genes common with genes identified in TIGIT^+^KLRG1^+^ T_EX_ as visualized in a protein-protein interaction network and were functionally similar (**Supplementary Figure 6B**).

Given the consistency of increased EOMES signature across disease settings and the correlation with TIGIT^+^KLRG1^+^ protein expression (**Figure 2**), we predicted that the increased EOMES signature in non-risk RA HLA subjects would also be reflected at the protein level. For this experiment, we measured the frequency of EOMES-associated TIGIT^+^KLRG1^+^ T_EX_ in CMV-negative age- and sex-matched HC and RA subjects selected for high versus low EOMES signature, defined by upper and lower terciles (**Table 1**). We did not observe differences in the frequency of EOMES-associated TIGIT^+^KLRG1^+^ T_EX_ between HC and RA subjects; nor were TIGIT^+^KLRG1^+^ T_EX_ functionally different (**Supplementary Figure 7**) as assessed by similarly low IFNγ production. However, there was a significant increase in TIGIT^+^KLRG1^+^ T_EX_ abundance in the non-risk RA HLA subjects as compared with risk RA HLA subjects (**Figure B**). Thus, these data suggest the autoimmune-associated RA HLA genotype or linked genes contributes to variation in the frequency of TIGIT^+^KLRG1^+^ T_EX_ in a cohort of HC and RA subjects.

### TIGIT^+^KLRG1^+^ T_EX_ are increased selectively in RA HLA risk subjects treated with abatacept (CTLA4Ig)

DR4 is a common risk allele between RA and T1D (28) and is associated with better outcome in a clinical trial of teplizumab (anti-CD3) therapy in individuals at risk for T1D (15). Given the association of T_EX_ with better response to therapy in autoimmune disease (12, 13, 15), we explored the relationship between TIGIT^+^KLRG1^+^ T_EX_ frequency and HLA risk alleles in the setting of immune interventions leveraging recent clinical trials. We first asked whether TIGIT^+^KLRG1^+^ T_EX_ are selectively modulated in DR4 T1D subjects, examining the teplizumab (anti-CD3) trial in individuals at risk for T1D since DR4 was previously identified as a weak correlate of response (15). We found a significant increase in TIGIT^+^KLRG1^+^ T_EX_ among DR4 risk subjects (*P* = 0.0033), but not DR4 non-risk subjects (*P* = 0.2650) (**Figure 5A**). Note, CMV seropositivity and mean age did not differ between DR4 risk and non-risk subjects. Thus, we link the previous DR4 association with response to a selective increase in TIGIT^+^KLRG1^+^ T_EX_ in DR4 subjects.

We analyzed CyTOF data from the Early AMPLE trial (ClinicalTrials.gov: NCT02557100), a randomized, head-to-head, single-blind study comparing abatacept (CTLA4Ig) and adalimumab (anti-TNF) in new-onset RA (16). The results from this trial in biologic naïve patients demonstrated a superior response in the abatacept arm that was more pronounced in subjects who carried the shared epitope alleles (HLA DR1, DR4, DR10) (16). Here, we examined TIGIT^+^KLRG1^+^ T_EX_ in the abatacept-treated group based on risk and non-risk RA HLA as defined in **Figure 4**. We did not find an increase in TIGIT^+^KLRG1^+^ T_EX_ with treatment across all subjects but there was notable heterogeneity. When stratifying by RA HLA risk, we observed a significant increase in the frequency of TIGIT^+^KLRG1^+^ T_EX_ in risk RA HLA subjects (*P* = 0.0043), but not non-risk RA HLA subjects (*P* = 0.1250) following treatment with abatacept (**Figure 5B**). In contrast, there was no change in the frequency of TIGIT^+^KLRG1^+^ T_EX_ in RA HLA risk subjects after adalimumab treatment in either risk or non-risk RA HLA subjects (**Figure 5C**). Mean age of RA HLA risk groups did not differ in either study. Collectively, these findings suggest that TIGIT^+^KLRG1^+^ T_EX_ frequency depends, in part, on HLA risk alleles and may be modulated by some immunotherapies.

## Discussion

T_EX_ are clearly associated with worse outcome in chronic viral infection and cancer (1), yet the opposing association of reduced T_EX_ with autoimmunity is more nuanced. For example, reduced T_EX_ have been associated with disease progression or severity (8, 10, 11) but not disease onset; in T1D, the frequency of T_EX_ does not discriminate HC from T1D, only rate of disease progression (10). Here, we associate reduced T_EX_ with RA HLA risk alleles in both HC and RA subjects, linking T_EX_ to predisposition to autoimmunity. In addition, co-stimulation blockade selectively increased T_EX_ in risk RA HLA subjects, suggesting this risk phenotype may be modulated with therapy. These findings may help determine who may respond best to T_EX_ augmenting therapies.

Reduced T_EX_ in HC and RA subjects carrying RA HLA risk alleles was enabled by identification of markers (TIGIT and KLRG1), which together broadly defined dysfunctional CD8 T cells across disease cohorts. The foundation of this observation lies in the EOMES transcriptional signature that we first defined and associated with TIGIT^+^ KLRG1^+^ CD8 T cells in T1D responders to teplizumab (anti-CD3) therapy (12) and here extended to HC, cancer, and chronic viral infection. EOMES has long been associated with T_EX_ when expressed at high levels in combination with other T_EX_-associated genes (29, 30) and is a common feature of multiple T_EX_ signatures (31–35), in which high levels of nuclear EOMES drives PD-1 expression (36), a common inhibitory receptor of T_EX_. Moreover, one of the co-expressed genes within the TIGIT^+^KLRG1^+^ EOMES signature is TOX which is a transcription factor known to promote T_EX_ differentiation, phenotype, and persistence (37, 38). Thus, TIGIT and KLRG1 surface co-expression broadly define T_EX_. However, it should be noted that this population broadly defines T_EX_ with different degrees of exhaustion suggesting that some subsets of TIGIT^+^KRLG1^+^ cells may be more exhausted than others (e.g. early and late memory) and is limited to application in humans since KLRG1 expression dynamics and association with T_EX_ differ in mice (39, 40).

The RA HLA risk association with lower TIGIT^+^KLRG1^+^ T_EX_ is unique in two ways. First, to our knowledge, this is the first linkage of an autoimmune-associated risk allele and T_EX_. HLA associations in RA have suggested involvement of antibody and CD4 T cell responses to date, not CD8 T cells (41). Although EOMES (42) and CD8 T cell differentiation states (43) have been linked to autoimmune-associated SNPs, association with RA HLA alleles has not previously been described. We suggest that the robust RA HLA association with T_EX_ that we identified was due to our experimental design, which used a broad definition of T_EX_ (as opposed to T_EX_ subsets), built from the observation that age and CMV seropositivity are not the only factors that contribute to increased TIGIT^+^KLRG1^+^ T_EX_, as well as the risk and non-risk RA HLA groups being matched for disease co-factors including age and stage of disease. Second, reduced T_EX_ are associated with a risk allele, not disease progression. This suggests that reduced T_EX_ in risk RA HLA subjects may play a role in autoimmune susceptibility as well as contributing to faster progression and increased severity (7, 9, 10). Thus, while antigen is a main driver of exhaustion, additional factors may reduce the frequency of T_EX_ including young age, a lack of environmental exposures (e.g., CMV seropositivity), and RA HLA risk alleles.

We identified a CD8 T cell subset that is associated with a Class II HLA allele. This is unusual since HLA Class II associations directly implicate a role for antigen-presenting cells and CD4 T cell help. For example, autoimmune-associated HLA alleles in RA and T1D are associated with the presence of specific autoantibodies (44). However, indirect linkage of T cell help and potential CD8 responses is not unprecedented; reduced autoantibody responses to specific islet antigens in T1D have been associated with the Class I HLA*24 allele (45). Our findings from therapeutic intervention also support an indirect influence of HLA on T_EX_ frequency. The fact that T_EX_ also increase in some non-risk RA HLA subjects, suggests that abatacept is not a driver of T_EX_, but instead, it influences factors that may promote expansion of T_EX_. Abatacept is known to block APC-CD4 T cell interactions resulting in reduced CD4 helper cells across multiple autoimmune diseases (46–51). Also, teplizumab (anti-CD3) therapy can result in T cell receptor activation without co-stimulation, which may limit CD4 T cell help. It has been shown that reduced T cell help can augment T_EX_ in other contexts (8, 52, 53). Further studies are needed to dissect the potential role of CD4 T cell help on T_EX_ in risk RA HLA subjects.

The HLA locus is complex and co-factors differ across diseases, leaving some questions. Unlike in HC and RA, reduced TIGIT^+^KLRG1^+^ T_EX_ were not associated with T1D HLA DR4 risk alleles at baseline in individuals with T1D. T1D shares some HLA risk alleles with RA including DRB1*0401, 0404, and 0405 but is uniquely associated with DRB1*0402 with an odds ratio higher than 8 (44). In addition, while the RA HLA-T_EX_ association is recapitulated in baseline samples from abatacept- (CTLA4Ig) treated RA subjects, it was not in the adalimumab (anti-TNF) RA treatment cohort; although, this may be due to higher baseline T_EX_ proportions; T_EX_ were significantly higher (*P* = 0.0036) at baseline in adalimumab- as compared to abatacept-treated RA subjects. Together these data suggest that the TIGIT^+^KLRG1^+^ T_EX_ association with HLA is not absolute and T1D-specific disease-related co-factors (e.g., age, stage of disease) may contribute to the lack of an RA HLA-T_EX_ association that is found in HC and RA. Alternatively, T_EX_ may be associated with an HLA linked gene that is less prevalent in T1D. These results justify a focused and larger follow-up study powered to address individual HLAs.

There are several limitations to this study. By focusing on a broad definition of T_EX_, we were not able determine associations with early, partial, or late T_EX_, however, based on the variability in the degree of reduced function, the TIGIT^+^KLRG1^+^ T_EX_ population is likely heterogeneous. We lack validation of the selective augmentation of T_EX_ in abatacept-treated RA subjects and do not have access to samples to ask about the transient or persistent nature of these increases. Identifying clinical correlates of immune response in the Early AMPLE trial (18) was challenging since the majority of subjects responded to abatacept. Thus, our studies do not support or discount the possibility that increasing T_EX_ with therapy improves outcome (decreases disease activity, ACPA or rheumatoid factor levels) in RA as has been shown in T1D with teplizumab (anti-CD3) therapy (12, 13). Moreover, the impact of abatacept may be subtle, as an EOMES signature of response was not found in individuals with T1D treated with abatacept; although, this could also be due to the timing of sampling (54). Nonetheless, some studies do suggest that modulating T_EX_ may influence RA disease outcome; immune checkpoint blockade reduces T_EX_ and can result in onset of RA (55, 56) and a reduction of CD28^-^ T cells (that may include T_EX_) has been associated with clinical response to abatacept (20).

In summary, we demonstrate that increased autoimmune genetic risk is associated with lower levels of hypofunctional TIGIT^+^KLRG1^+^ T_EX_. TIGIT^+^KLRG1^+^ T_EX_ in RA HLA risk subjects can be selectively augmented by treatment with abatacept (CTLA4Ig) in RA and by teplizumab (anti-CD3) in T1D. More broadly, these studies demonstrate that variability in T_EX_ frequencies is not only associated with disease severity or progression, but also disease risk, and lower levels of T_EX_ may be used as a selection criterion for treatments that augment T_EX_.

## Data availability statement

The RNA-seq data is available through the GEO Repository (GSE216680). Flow cytometry data is accessible through TrialNet, ITN or Allen Institute portals or IMPORT. All other data are available in the main text or the **Supplementary Materials**.

## Ethics statement

The studies involving humans were approved by Benaroya Research Institute (IRB07109) and VA Puget Sound Health Care System (MIRB#00755). The studies were conducted in accordance with the local legislation and institutional requirements. The participants provided their written informed consent to participate in this study.

## Author contributions

SL: Conceptualization, Data curation, Formal analysis, Funding acquisition, Investigation, Methodology, Project administration, Resources, Supervision, Validation, Visualization, Writing – original draft, Writing – review & editing. VM: Data curation, Formal analysis, Investigation, Methodology, Visualization, Writing – review & editing. BJ: Data curation, Formal analysis, Investigation, Methodology, Visualization, Writing – review & editing. VW: Data curation, Formal analysis, Investigation, Methodology, Visualization, Writing – review & editing. AY: Formal analysis, Visualization, Writing – review & editing. AMH: Writing – original draft, Writing – review & editing. SP: Formal analysis, Methodology, Visualization, Writing – review & editing. JT: Formal analysis, Investigation, Methodology, Visualization, Writing – review & editing. BF: Formal analysis, Investigation, Visualization, Writing – review & editing. AW: Formal analysis, Investigation, Methodology, Visualization, Writing – review & editing. MT: Formal analysis, Investigation, Visualization, Writing – review & editing. KL: Formal analysis, Investigation, Methodology, Visualization, Writing – review & editing. HU: Formal analysis, Investigation, Visualization, Writing – review & editing. CS: Investigation, Resources, Writing – review & editing. BN: Investigation, Resources, Writing – review & editing. ATH: Investigation, Resources, Writing – review & editing. TT: Investigation, Resources, Writing – review & editing. AS: Investigation, Resources, Writing – review & editing. MM: Investigation, Resources, Writing – review & editing. NR: Investigation, Resources, Writing – review & editing. VK: Investigation, Resources, Writing – review & editing. JL: Investigation, Resources, Writing – review & editing. PL: Conceptualization, Data curation, Formal analysis, Investigation, Methodology, Visualization, Writing – review & editing. JB: Conceptualization, Data curation, Formal analysis, Funding acquisition, Investigation, Methodology, Project administration, Resources, Supervision, Validation, Visualization, Writing – original draft, Writing – review & editing.
