# Supplementary material for: Abatacept increases T cell exhaustion in early RA individuals who carry HLA risk alleles
Source: Front Immunol. 2024 Apr 8;15:1383110. doi: 10.3389/fimmu.2024.1383110 (PMC11033422; doi:10.3389/fimmu.2024.1383110)
Supplement: Supplementary file 2 [file Presentation_1.pptx]

## Slide 1
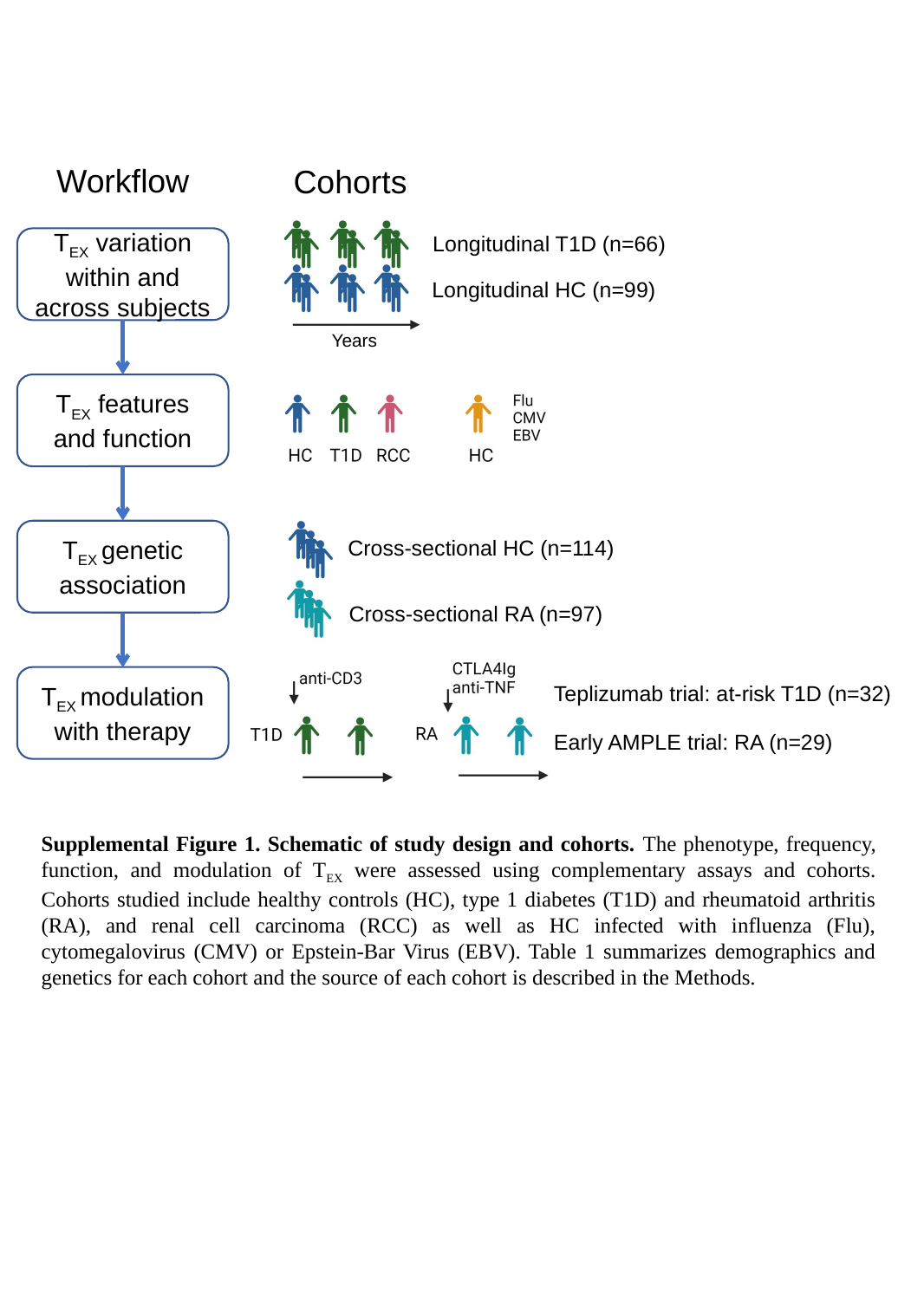

Workflow
TEX variation within and across subjects
TEX features and function
TEX genetic association
TEX modulation with therapy
Cohorts
Years
Longitudinal T1D (n=66)
Longitudinal HC (n=99)
Cross-sectional HC (n=114)
Cross-sectional RA (n=97)
Teplizumab trial: at-risk T1D (n=32)
Early AMPLE trial: RA (n=29)
Supplemental Figure 1. Schematic of study design and cohorts. The phenotype, frequency, function, and modulation of TEX were assessed using complementary assays and cohorts. Cohorts studied include healthy controls (HC), type 1 diabetes (T1D) and rheumatoid arthritis (RA), and renal cell carcinoma (RCC) as well as HC infected with influenza (Flu), cytomegalovirus (CMV) or Epstein-Bar Virus (EBV). Table 1 summarizes demographics and genetics for each cohort and the source of each cohort is described in the Methods.

## Slide 2
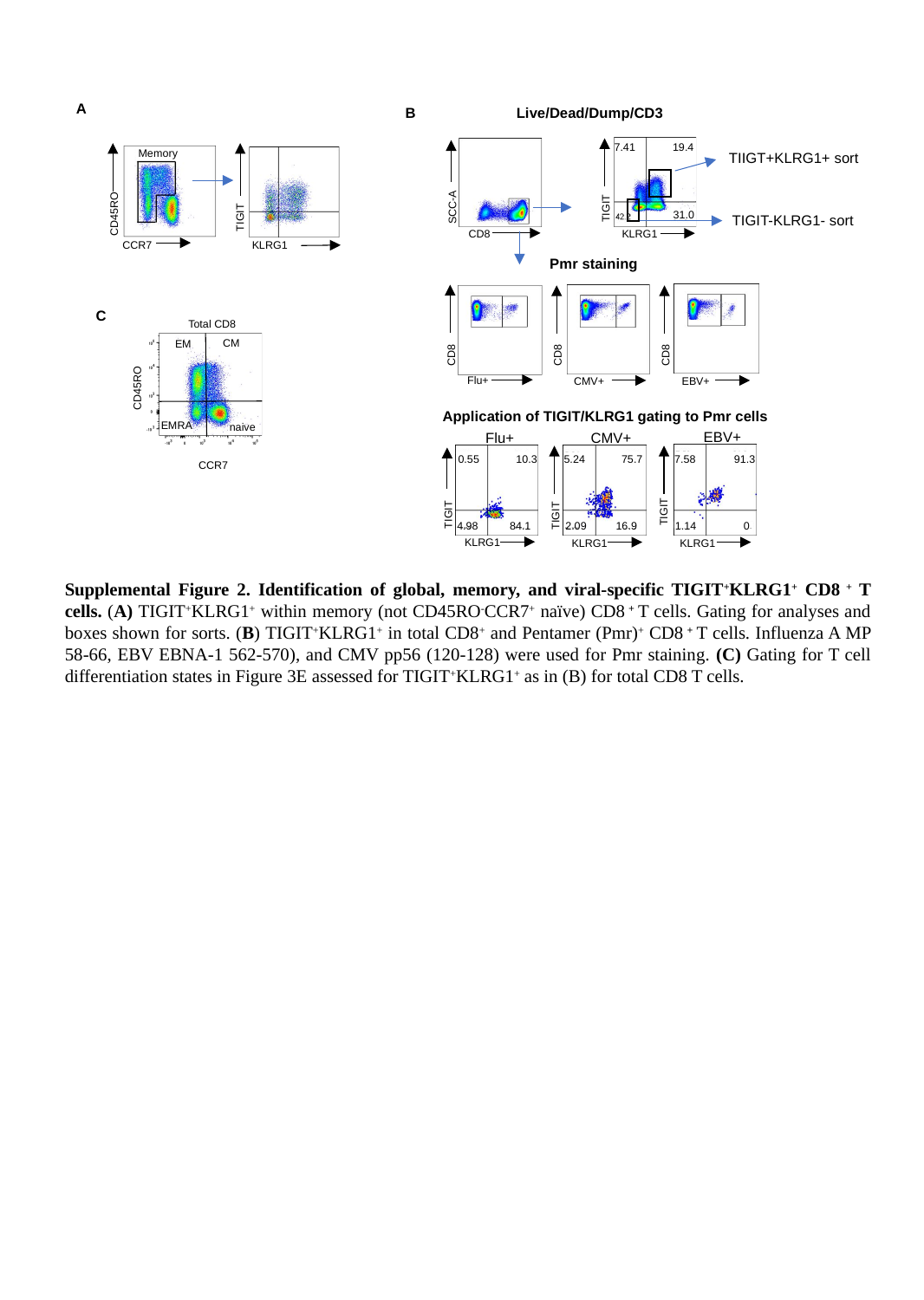

A
B
Live/Dead/Dump/CD3
7.41
19.4
Memory
TIIGT+KLRG1+ sort
SCC-A
TIGIT
CD45RO
31.0
TIGIT
TIGIT-KLRG1- sort
CD8
KLRG1
CCR7
KLRG1
Pmr staining
CD8
CD8
CD8
Flu+
CMV+
EBV+
Application of TIGIT/KLRG1 gating to Pmr cells
EBV+
Flu+
CMV+
0.55
10.3
5.24
75.7
7.58
91.3
TIGIT
TIGIT
TIGIT
4.98
84.1
2.09
16.9
1.14
0
KLRG1
KLRG1
KLRG1
Supplemental Figure 2. Identification of global, memory, and viral-specific TIGIT+KLRG1+ CD8 + T cells. (A) TIGIT+KLRG1+ within memory (not CD45RO-CCR7+ naïve) CD8 + T cells. Gating for analyses and boxes shown for sorts. (B) TIGIT+KLRG1+ in total CD8+ and Pentamer (Pmr)+ CD8 + T cells. Influenza A MP 58-66, EBV EBNA-1 562-570), and CMV pp56 (120-128) were used for Pmr staining. (C) Gating for T cell differentiation states in Figure 3E assessed for TIGIT+KLRG1+ as in (B) for total CD8 T cells.
C
Total CD8
CM
EM
CD45RO
EMRA
naive
CCR7

## Slide 3
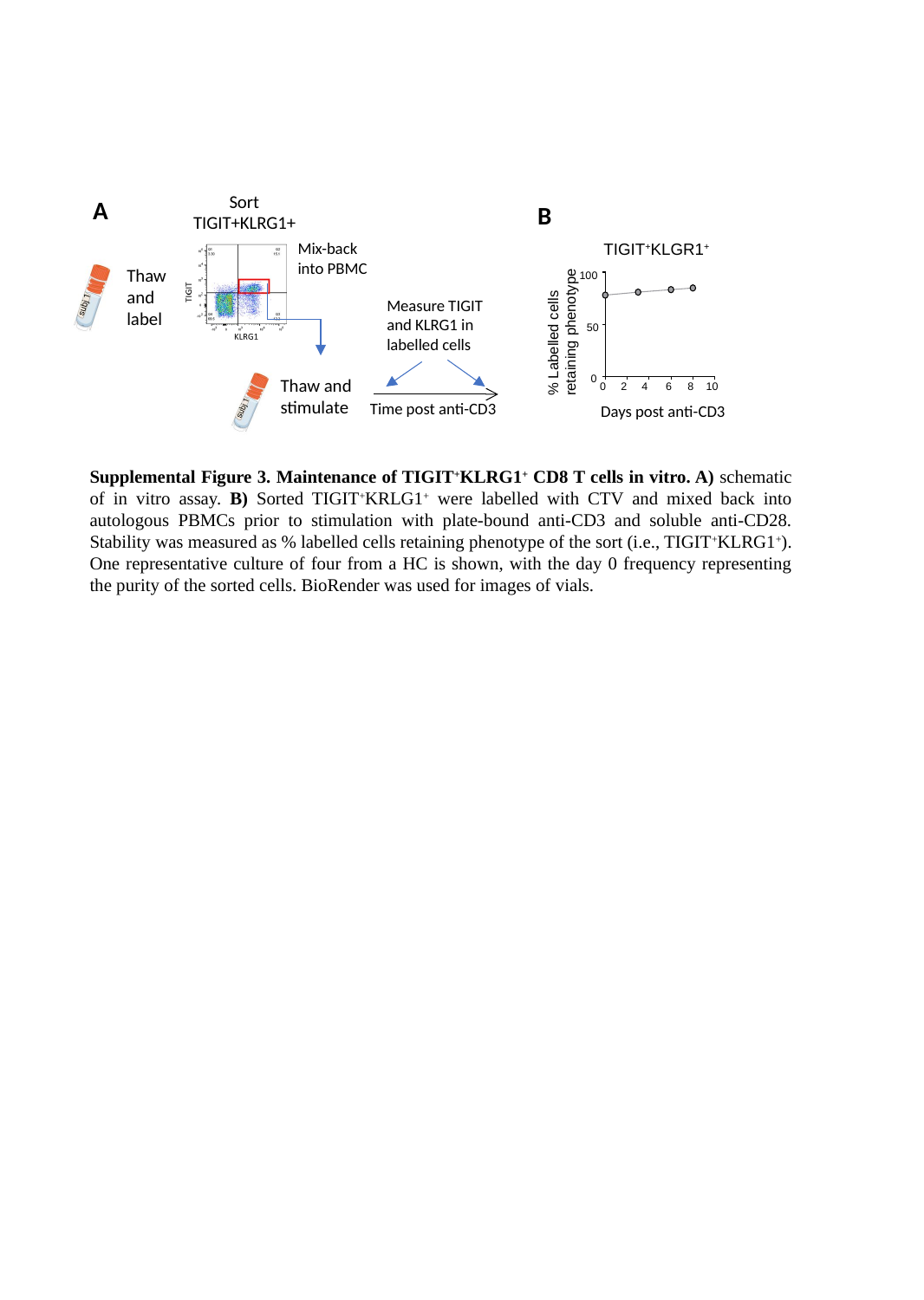

Sort TIGIT+KLRG1+
A
B
TIGIT+KLGR1+
100
50
0
% Labelled cells retaining phenotype
0
2
4
10
6
8
Mix-back into PBMC
Thaw and label
Measure TIGIT and KLRG1 in labelled cells
Thaw and stimulate
Time post anti-CD3
Days post anti-CD3
Supplemental Figure 3. Maintenance of TIGIT+KLRG1+ CD8 T cells in vitro. A) schematic of in vitro assay. B) Sorted TIGIT+KRLG1+ were labelled with CTV and mixed back into autologous PBMCs prior to stimulation with plate-bound anti-CD3 and soluble anti-CD28. Stability was measured as % labelled cells retaining phenotype of the sort (i.e., TIGIT+KLRG1+). One representative culture of four from a HC is shown, with the day 0 frequency representing the purity of the sorted cells. BioRender was used for images of vials.

## Slide 4
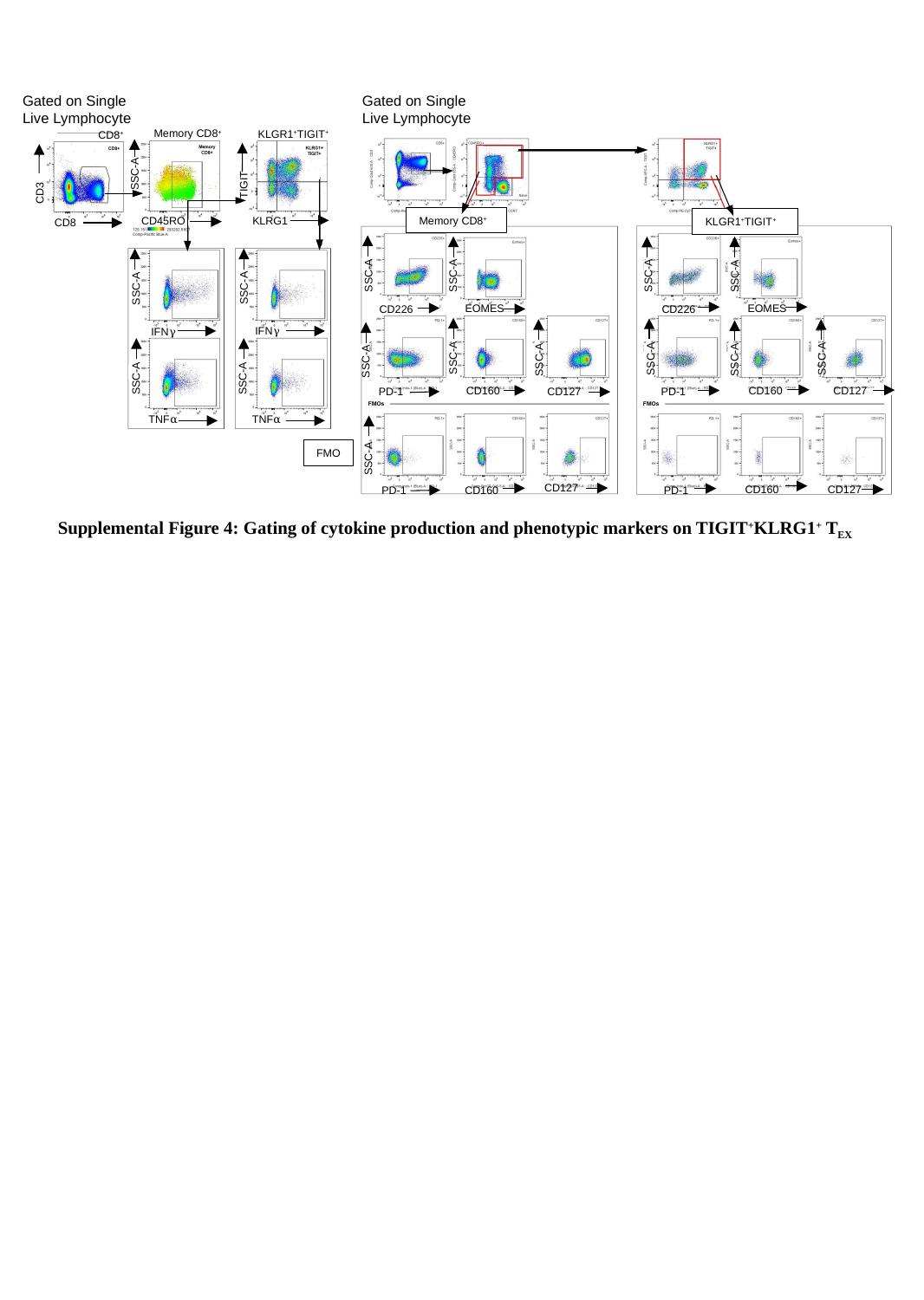

Gated on Single
Live Lymphocyte
Gated on Single
Live Lymphocyte
Memory CD8+
KLGR1+TIGIT+
CD8+
SSC-A
TIGIT
CD3
Memory CD8+
CD45RO
KLRG1
KLGR1+TIGIT+
CD8
SSC-A
SSC-A
SSC-A
SSC-A
SSC-A
SSC-A
EOMES
EOMES
CD226
CD226
IFNγ
IFNγ
SSC-A
SSC-A
SSC-A
SSC-A
SSC-A
SSC-A
SSC-A
SSC-A
CD127
CD160
CD160
CD127
PD-1
PD-1
TNFα
TNFα
SSC-A
FMO
CD127
CD160
CD127
PD-1
CD160
PD-1
Supplemental Figure 4: Gating of cytokine production and phenotypic markers on TIGIT+KLRG1+ TEX

## Slide 5
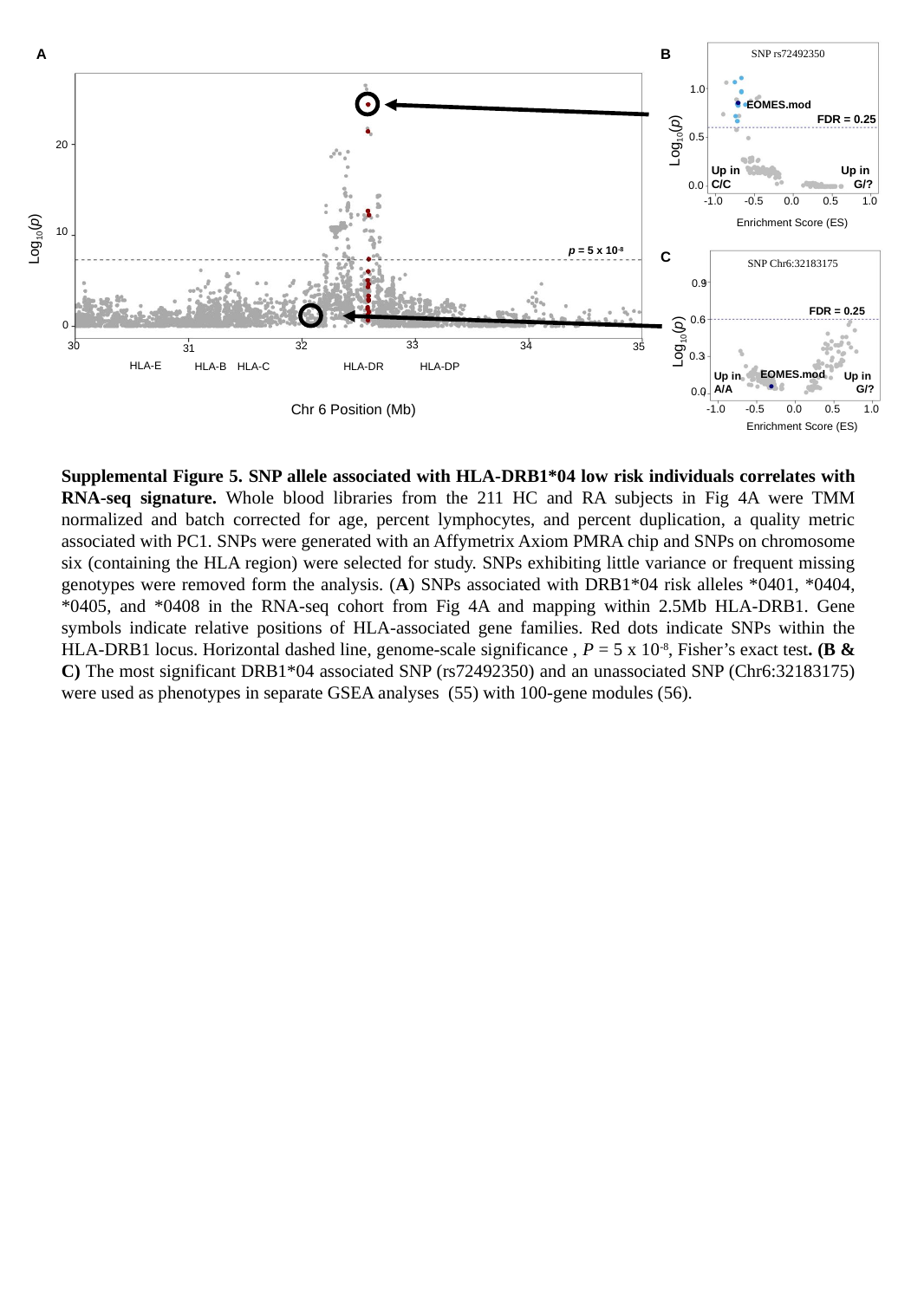

A
B
1.0
0.5
0.0
EOMES.mod
Log10(p)
FDR = 0.25
20
10
0
Log10(p)
Up in C/C
Up in
G/?
-1.0
-0.5
0.0
0.5
1.0
Enrichment Score (ES)
p = 5 x 10^-8^
C
0.9
FDR = 0.25
0.6
0.3
0.0
Log10(p)
33
34
30
32
31
35
HLA-E
HLA-B
HLA-C
HLA-DP
HLA-DR
EOMES.mod
Up in
A/A
Up in
G/?
Chr 6 Position (Mb)
-1.0
-0.5
0.0
0.5
1.0
Supplemental Figure 5. SNP allele associated with HLA-DRB1*04 low risk individuals correlates with RNA-seq signature. Whole blood libraries from the 211 HC and RA subjects in Fig 4A were TMM normalized and batch corrected for age, percent lymphocytes, and percent duplication, a quality metric associated with PC1. SNPs were generated with an Affymetrix Axiom PMRA chip and SNPs on chromosome six (containing the HLA region) were selected for study. SNPs exhibiting little variance or frequent missing genotypes were removed form the analysis. (A) SNPs associated with DRB1*04 risk alleles *0401, *0404, *0405, and *0408 in the RNA-seq cohort from Fig 4A and mapping within 2.5Mb HLA-DRB1. Gene symbols indicate relative positions of HLA-associated gene families. Red dots indicate SNPs within the HLA-DRB1 locus. Horizontal dashed line, genome-scale significance , P = 5 x 10^-8^, Fisher’s exact test. (B & C) The most significant DRB1*04 associated SNP (rs72492350) and an unassociated SNP (Chr6:32183175) were used as phenotypes in separate GSEA analyses (55) with 100-gene modules (56).
SNP rs72492350
SNP Chr6:32183175
Enrichment Score (ES)

## Slide 6
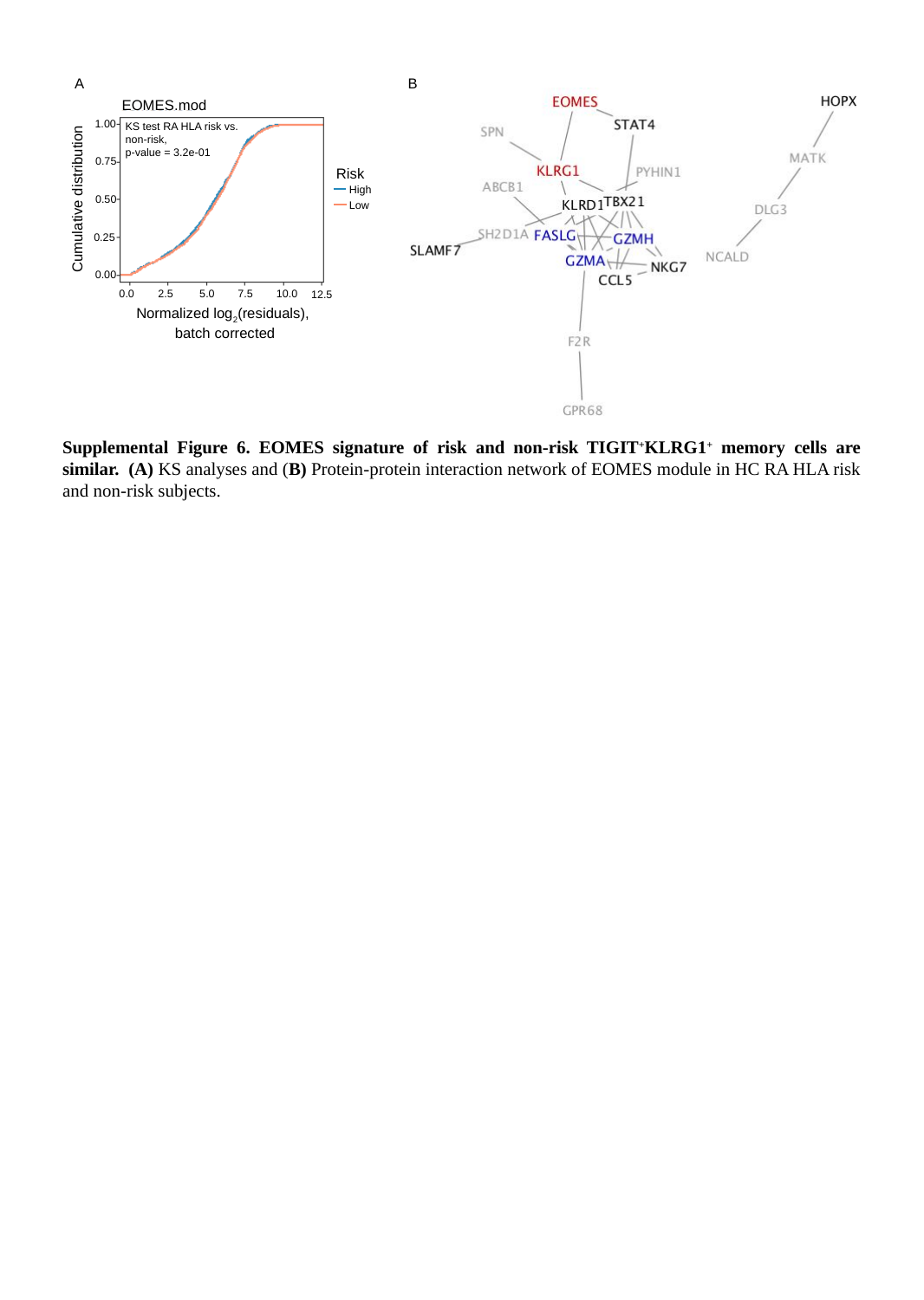

A
B
EOMES.mod
1.00
0.75
0.50
0.25
0.00
KS test RA HLA risk vs. non-risk,
p-value = 3.2e-01
Risk
Cumulative distribution
High
Low
0.0
2.5
5.0
7.5
10.0
12.5
Normalized log2(residuals),
batch corrected
Supplemental Figure 6. EOMES signature of risk and non-risk TIGIT+KLRG1+ memory cells are similar. (A) KS analyses and (B) Protein-protein interaction network of EOMES module in HC RA HLA risk and non-risk subjects.

## Slide 7
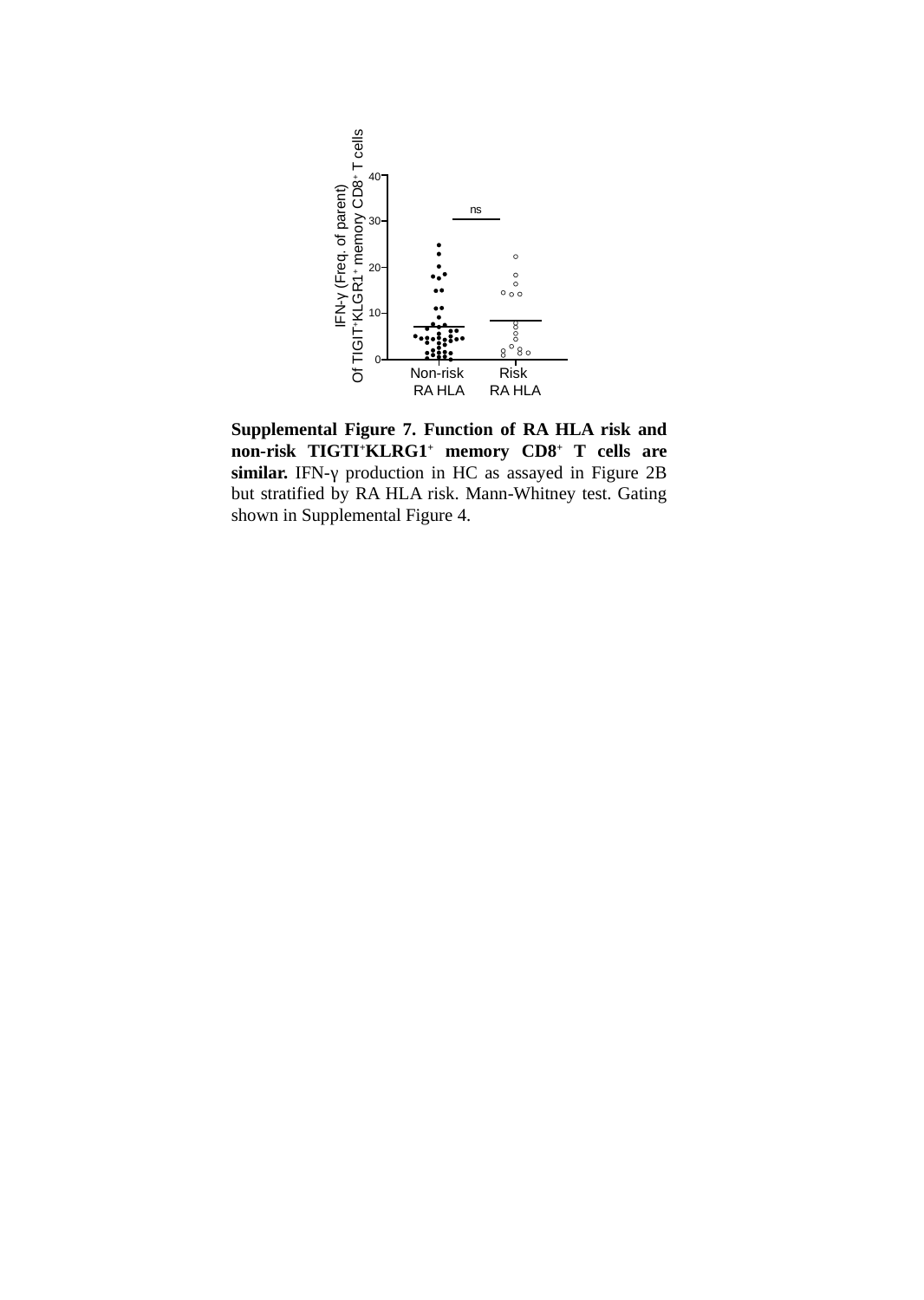

40
30
20
10
0
IFN-γ (Freq. of parent)
Of TIGIT+KLGR1+ memory CD8+ T cells
Non-risk
RA HLA
Risk
RA HLA
Supplemental Figure 7. Function of RA HLA risk and non-risk TIGTI+KLRG1+ memory CD8+ T cells are similar. IFN-γ production in HC as assayed in Figure 2B but stratified by RA HLA risk. Mann-Whitney test. Gating shown in Supplemental Figure 4.

## Slide 8
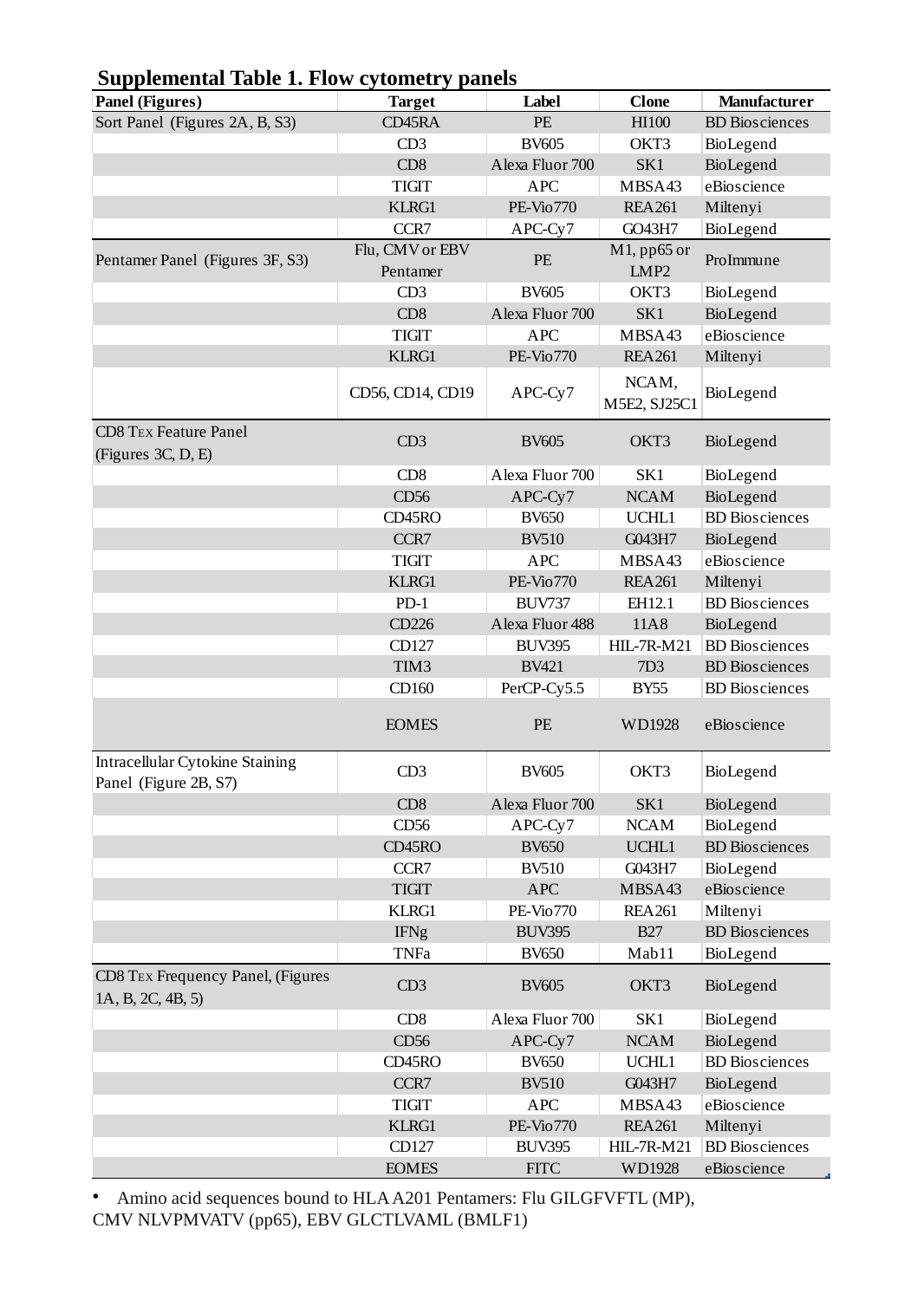

Supplemental Table 1. Flow cytometry panels
Amino acid sequences bound to HLA A201 Pentamers: Flu GILGFVFTL (MP),
CMV NLVPMVATV (pp65), EBV GLCTLVAML (BMLF1)

## Slide 9
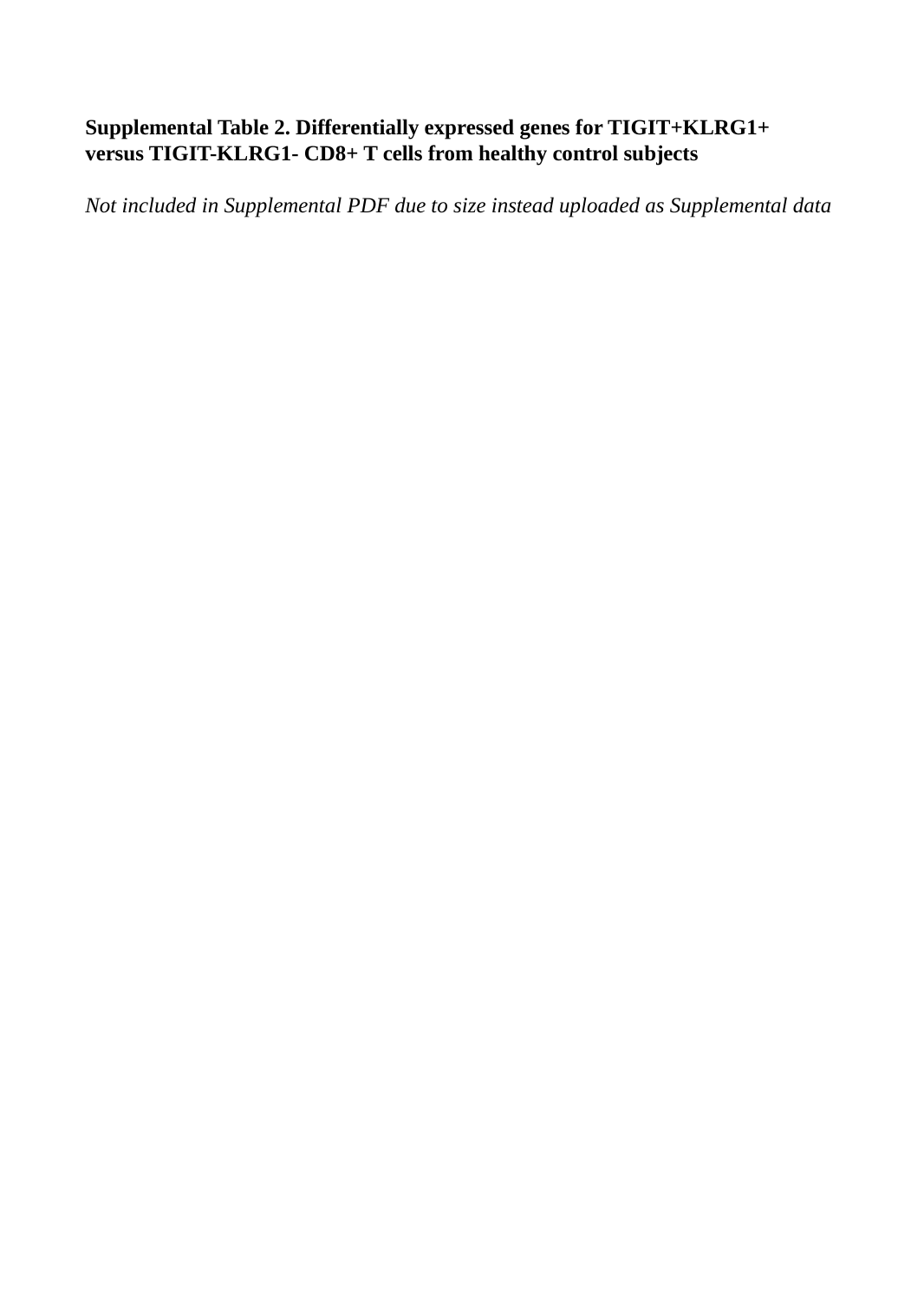

Supplemental Table 2. Differentially expressed genes for TIGIT+KLRG1+
versus TIGIT-KLRG1- CD8+ T cells from healthy control subjects
Not included in Supplemental PDF due to size instead uploaded as Supplemental data

## Slide 10
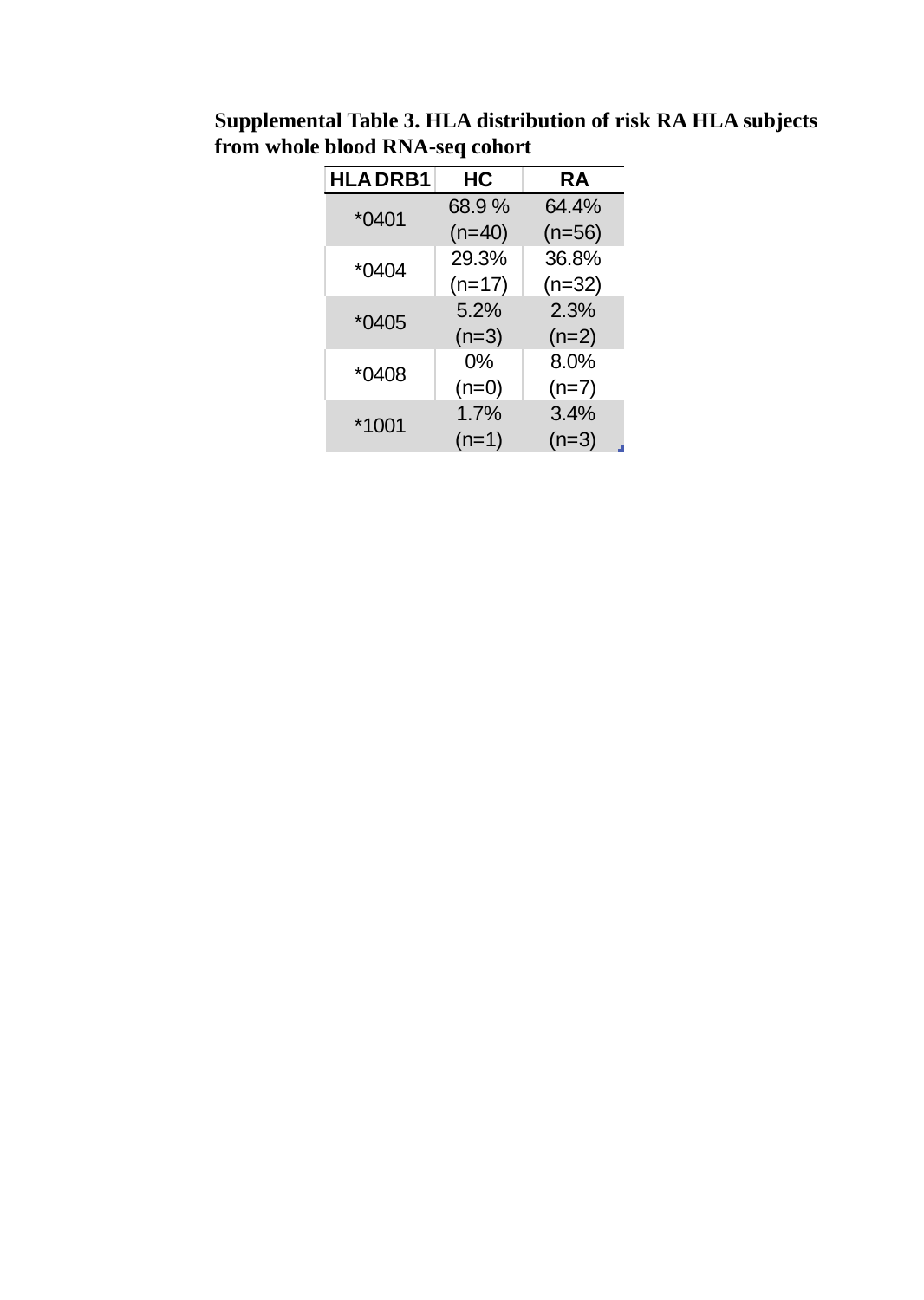

Supplemental Table 3. HLA distribution of risk RA HLA subjects
from whole blood RNA-seq cohort

## Slide 11
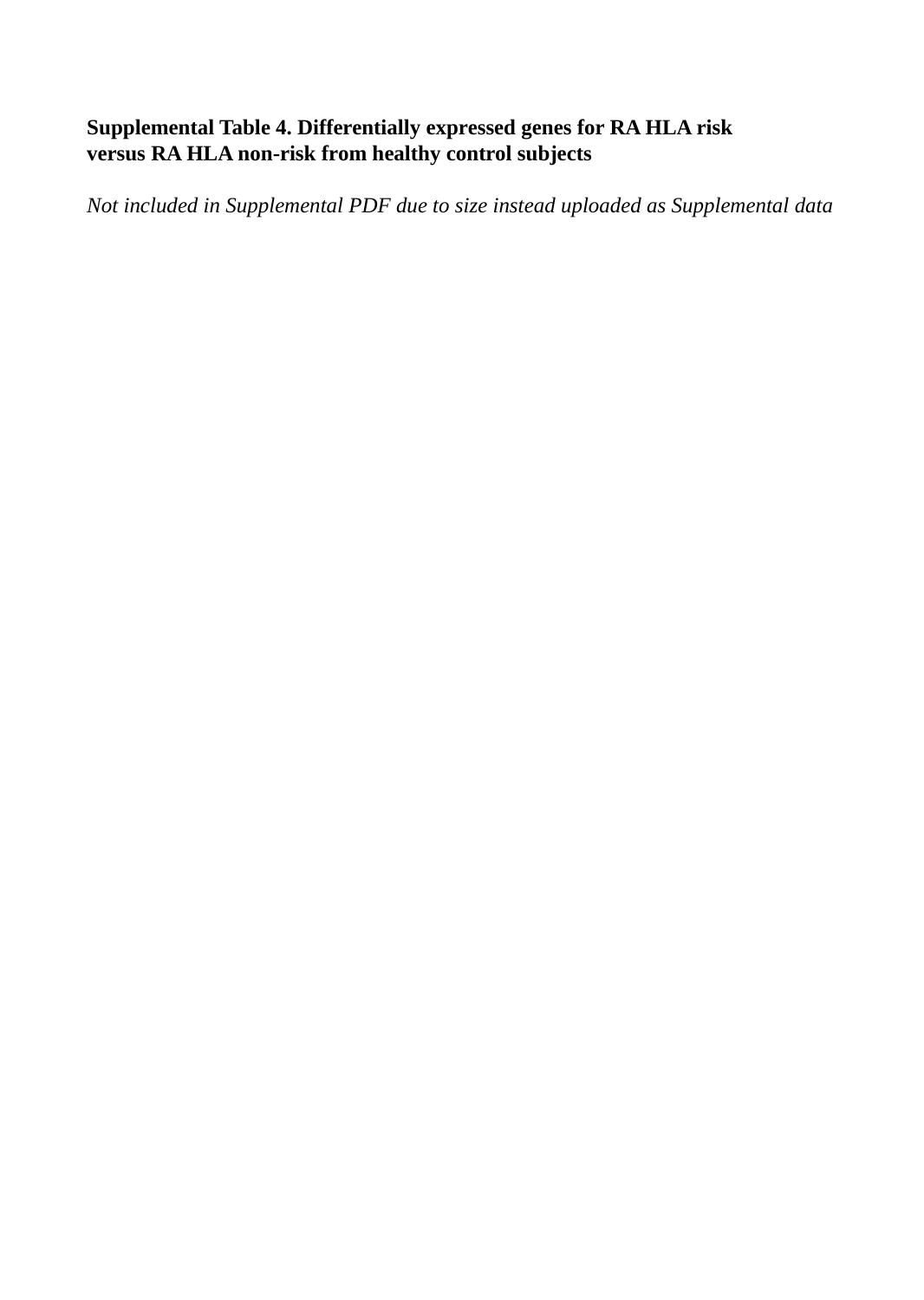

Supplemental Table 4. Differentially expressed genes for RA HLA risk
versus RA HLA non-risk from healthy control subjects
Not included in Supplemental PDF due to size instead uploaded as Supplemental data
